# Tart cherries (*Prunus cerasus*) and metabolic health in overweight and obesity: evidence from preclinical and clinical studies

**DOI:** 10.3389/fnut.2026.1828579

**Published:** 2026-05-15

**Authors:** Zuzanna Koziara, Maria Vittoria Micioni Di Bonaventura, Carlo Cifani

**Affiliations:** School of Pharmacy, Pharmacology Unit, University of Camerino, Camerino, Italy

**Keywords:** anthocyanins, cherry juice, cherry powder, metabolic syndrome, oxidative stress, phytochemicals, sour cherry

## Abstract

Overweight and obesity remain major global health challenges and are closely associated with metabolic disturbances, including dyslipidemia, insulin resistance, hypertension, and chronic low-grade inflammation. Dietary patterns rich in fruits and vegetables contribute to metabolic health, partly due to their complex matrix of bioactive compounds. Tart cherries (*Prunus cerasus*, TC) are widely consumed in fresh and processed forms and have attracted attention for their potential benefits in obesity-related conditions. This review critically summarizes current evidence from *in vitro*, animal, and human studies investigating the effects of tart cherries and their derived products in overweight and obese individuals (BMI ≥ 25 kg/m^2^). Animal studies consistently reported anti-inflammatory, antioxidant, and metabolic improvements following tart cherry supplementation. In humans, the evidence suggested potential benefits for blood pressure and selected inflammatory markers, whereas findings regarding lipid profile, glucose metabolism, and body composition remain inconsistent. Overall, tart cherries may favorably influence metabolic and vascular processes associated with obesity; however, heterogeneity in study design, dosage, and product form limits definitive conclusions. Further research should focus on identifying the key bioactive components, understanding their bioavailability and mechanisms of action, and determining which populations may benefit most from TC-based interventions.

## 1 Introduction

Nowadays, overweight and obesity, defined as excessive fat deposition, constitute a global epidemic. According to World Health Organization (WHO), in 2022, approximately 2.5 billion adults were affected by overweight (BMI ≥ 25 kg/m^2^), including 890 million who met the criteria for obesity (BMI ≥ 30 kg/m^2^) (1). These conditions reduce quality of life and are associated with numerous health complications, including cardiovascular disease, type 2 diabetes, hyperlipidemia, hypertension, various types of cancer, and chronic inflammation (2). Overweight and obesity also generate significant economic costs, estimated at 2.19% of global gross domestic product (GDP) in 2019, with a per capita burden of US$542 in Europe and US$872 in the Americas (3). Although several anti-obesity drugs are currently available, a healthy lifestyle, based on a normocaloric diet and adequate physical activity, resulting in energy balance, remains the cornerstone of prevention (4).

One of the foundations of a healthy diet is the consumption of fruits and vegetables, which, due to their fiber and antioxidants content, contribute to the maintenance of proper body weight (BW) (5, 6). Particular attention has been given to plant-derived foods rich in bioactives, including anthocyanins, believed to reduce inflammation, oxidative stress, and modulate gut microbiota (7–9). Tart (sour) cherries (*Prunus cerasus*; TC) are particularly rich in these compounds. Cherries rank among the earliest cultivated fruits, with archaeological findings of cherry stones dating from 5,000–4,000 BCE indicating their use by humans. This long history of consumption, together with their increasing recognition as a functional food, supports their relevance in nutritional research. Nowadays, TC are widely cultivated in America, Asia, and Europe, with leading producers including the Russian Federation, Türkiye, and Poland, and are consumed in various dietary forms such as juices, preserves, and concentrates. Popular cultivars include ‘Montmorency’, ‘Balaton’, ‘Marasca’ and ‘Kütahya’ (10, 11). Owing to their chemical composition (described below), TC have been extensively studied for their health properties, focusing on their antioxidant and anti-inflammatory effects, as well as potential benefits for cardiovascular disease, diabetes, cancer, muscle recovery, gout, and sleep (12–16). Given these properties, TC have been proposed as a potential dietary component influencing metabolic processes relevant to overweight and obesity, which warrants future evaluation. Therefore, understanding the chemical composition of TC and the impact of processing on bioactive compounds is essential for interpreting their potential metabolic and anti-obesity effects. Despite the growing body of research, findings remain heterogeneous and sometimes inconsistent, particularly in human studies, highlighting the need for a critical synthesis of current evidence. To the best of the authors’ knowledge, this is the first comprehensive review focusing specifically on the effects of TC in overweight and obese individuals, based on both animal and human studies.

## 2 Chemical composition of tart cherries relevant to metabolic health

The health benefits of TC are related to their chemical composition. The physicochemical characteristics of the fruit depend on the cultivar, maturity, climatic conditions (e.g., temperature, light intensity, water availability), and pre- and postharvest treatment (13, 17–19). Compared with sweet cherries (*Prunus avium*), TC contain lower amounts of simple sugars (17). The main saccharides contributing to the fruit’s sweetness are glucose, fructose and sucrose. In 21 TC varieties grown in China, glucose content ranged from 6.4 to 12.2 g/100 g of fresh weight (FW), fructose from 3.0 to 7.1 g/100 g FW, and sucrose from 0.5 to 1.2 g/100 g FW (20).

Another parameter influencing consumer preference is acidity, which in the case of cherries is caused by organic acids, primarily malic acid. The average content of this compound in 21 cultivars of cherries grown in Poland was 1655.2 mg/100 g FW. In the case of other organic acids identified in TC, the average content reached 305.0, 9.4, 2.2 and 0.2 mg/100 g FW for malonic, oxalic, shikimic and fumaric acid, respectively (19). Moreover, TC contain vitamin A (1283.0 IU/100 g FW), vitamin C (10.0 mg/100 g FW), vitamin E (70 μg/100 g FW), *β*-carotene (770.0 μg/100 g FW), as well as potassium (250.9–453.9 mg/100 g FW), iron (0.9–3.0 mg/100 g FW), manganese (0.3–0.4 mg/100 g of dry weight (DW)), magnesium (111.8–126.7 mg/100 g DW), while the amount of fiber is relatively low (1.6 g/100 g FW) (20–22). Some studies indicated the presence of melatonin in Montmorency cherries at the level of 13.5 ng/g of tissue (23), and 12.3 ng/g DW (24).

The health-promoting properties of TC are largely attributed to their rich content of bioactives, including phenolic compounds with antioxidant properties. However, these effects cannot be linked to a single class of molecules, as other constituents and potential synergistic interactions within the food matrix may also play an important role (25). The action of above mentioned phytochemicals, together with endogenous antioxidants produced in the body, can prevent pathophysiological processes resulting from disruption of redox homeostasis (26). TC are particularly rich in anthocyanins, which are responsible for the intense red color of the fruit. Representative values and corresponding quantification approaches are summarized in Table 1 (18–20, 24, 27, 28). The main anthocyanins detected in TC were cyanidin 3-glucosylrutinoside, cyanidin 3-rutinoside, cyanidin 3-sophoroside, and cyanidin 3-glucoside, as consistently reported across studies, although their relative abundance varies depending on cultivar, processing, and analytical methodology (19, 22, 24, 27–30) Many studies have shown that anthocyanins have a beneficial effect on the cardiovascular system, and they are also characterized by anticancer, anti-inflammatory, anti-diabetic, anti-obesity, and neuroprotective properties, which have already been widely described in the literature (31–33).

**Table 1:** Anthocyanins content in tart cherries across studies from different countries.

Reference	Country	No. of analyzed cultivars	Anthocyanins content	Processing and quantification method
(18)	Croatia	5	315.0–476.0 mg/100 g FW	Frozen (−20 °C)–thawed, depitted, homogenized fruits; acidified methanol extraction + pH differential spectrophotometric assay (510/700 nm); results expressed as cyanidin-3-O-glucoside equivalents
(19)	Poland	21	18.0–131.3 mg/100 g FW	Frozen (−20 °C)–thawed, depitted, homogenized fruits; acidified methanol extraction + HPLC-PDA (520 nm); results expressed as cyanidin-3-O-glucoside equivalents
(20)	China	21	39.0–410.8 mg/100 g DW	Depitted, freeze-dried fruits, homogenized in liquid nitrogen and stored at −80 °C; acidified methanol extraction + pH differential spectrophotometric assay (520/700 nm); results expressed as cyanidin-3-glucoside equivalents
(24)	United States	2	53.3–174.1 mg/100 g DW	Frozen, depitted freeze-dried fruits; acidified methanol extraction + pH differential spectrophotometric assay (510/700 nm); results expressed as cyanidin-3-glucoside equivalents
(27)	Italy	3	27.8–80.4 mg/100 g	Frozen (−20 °C; flushed with nitrogen before), depitted fruits homogenized in liquid nitrogen; acidified methanol extraction + HPLC-DAD (518 nm); results expressed as cyanidin-3-glucoside equivalents
(28)	Hungary	5	109.0–261.0 mg/100 g FW	Frozen (−20 °C), depitted, homogenized fruits; acidified methanol extraction + SPE purification; results expressed as cyanidin-3-glucoside equivalents
			21.0–295.0 mg/100 g FW	pH differential spectrophotometric assay (530/700 nm); results expressed as cyanidin-3-glucoside equivalents

DW, dry weight; FW, fresh weight.

Cherries are also a source of other phenolic compounds, which, according to Wojdyło et al., can reach concentrations of 362.4–435.8 mg/100 g DW for flavanols, 54.3–120.5 mg/100 g DW for phenolic acids, 111.9–157.7 mg/100 g DW for flavonols and flavonones (22). In the group of flavanols, the most common compounds determined in TC were catechin, epicatechin, and procyanidins. In the case of phenolic acids, the main ones were neochlorogenic, chlorogenic, and p-coumaric acids, while flavonols were primarily represented by derivatives of quercetin, kaempferol, and isorhamnetin (19, 34–37), as consistently observed across studies, although their relative abundance varies depending on cultivar, processing, and analytical methodology. The mentioned phenolic compounds have a broad range of health-promoting effects, which have already been well documented (38–40). Detailed compound–activity relationships in TC have been comprehensively reviewed elsewhere (41), while Table 2 provides a summary of the TC composition.

**Table 2:** Key representative components of tart cherries.

Compound/ group of compounds	Content	Unit	Reference
Glucose	6.4–12.2	g/100 g FW	(20)
Fructose	3.0–7.1	g/100 g FW	(20)
Sucrose	0.5–1.2	g/100 g FW	(20)
Malic acid	1655.2	mg/100 g FW	(19)
Malonic acid	305.0	mg/100 g FW	(19)
Oxalic acid	9.4	mg/100 g FW	(19)
Shikimic acid	2.2	mg/100 g FW	(19)
Fumaric acid	0.2	mg/100 g FW	(19)
Vitamin A	1283.0	IU/100 g FW	(21)
Vitamin C	10.0	mg/100 g FW	(21)
Vitamin E	70.0	μg/100 g FW	(21)
*β*-carotene	770.0	μg/100 g FW	(21)
Potassium	250.9–453.9	mg/100 g FW	(20)
Iron	0.9–3.0	mg/100 g FW	(20)
Manganese	0.3–0.4	mg/100 g DW	(22)
Magnesium	111.8–126.7	mg/100 g DW	(22)
Fiber	1.6	g/100 g FW	(21)
Melatonin	13.5	ng/g of tissue	(23)
	12.3	ng/g DW	(24)
Anthocyanins	See Table 1		
Flavanols	362.4–435.8	mg/100 g DW	(22)
Phenolic acids	54.3–120.5	mg/100 g DW	(22)
Flavonols and flavanones	111.9–157.7	mg/100 g DW	(22)

DW, dry weight; FW, fresh weight.

The chemical composition of fruit changes with the degree of ripeness. During maturation, the content of anthocyanins in cherries increases and peaking in fully ripe fruit, while phenolic acids, vitamin C, and antioxidant activity tend to decrease (42–44). TC are most often consumed in processed form, including jams, juices, and as dried or frozen fruit (11). Industrial and culinary processing may affect the chemical composition of fruit and, consequently, their health properties. Studies show that TC juices retain high anthocyanin levels despite processing. Toydemir et al. have shown that cyanidin-3-(2G-glucosylrutinoside) in cherry nectar reached 87% of the original fruit content (45). In the case of juices obtained from Turkish cherry varieties, the content of anthocyanins was in the range of 350.0–633.5 mg/L (46), and for commercial juice subjected to freeze-drying it reached up to 20 mg/100 g of freeze-dried product (47). It turns out that storage has a greater effect on the loss of anthocyanins, because after 6 months of storing pasteurized juice at 20 °C, the content of these compounds decreased by 70–75% (48). A significantly higher loss of anthocyanins was observed in the case of cherry jams, which amounted to up to 79% in the case of the product from the Balaton cherry variety (49). Levaj et al. showed that the most unstable phenolic compounds during jam production were a derivative of caffeic acid and flavan-3-ols (35), while in the case of TC juice, hydroxycinnamic acids and quercetin derivatives turned out to be more stable compared to anthocyanins (48). In the case of other cherry products, frozen fruit had a higher content of anthocyanins, total phenolics and melatonin compared to dried fruit and cherry concentrate (24). Despite the various types of processing, the antioxidant capacity of the final products is usually quite well retained (24, 48, 49), but it may vary depending on the fruit variety (46). The results obtained by Cairone et al. indicate that the best way to preserve bioactive compounds in fruit is to freeze them immediately after harvesting (50).

## 3 Search strategy and study selection

A literature search was conducted using Google Scholar, PubMed, and Scopus between December 2024 and up to September 11, 2025, using combinations of keywords such as “tart cherry,” “sour cherry,” “*Prunus cerasus*,” “obesity,” “overweight,” and “metabolic health.” Original research articles, including *in vitro*, animal, and human studies, were considered. For human studies, only those involving participants with a BMI ≥ 25 kg/m^2^ were included. Studies focusing exclusively on isolated compounds (e.g., purified, single anthocyanins) rather than whole tart cherry products were excluded. Additional exclusion criteria included reviews, conference abstracts, and non-English articles. Studies were selected based on their relevance to the topic, with a focus on the metabolic effects. Given the narrative nature of this review, a formal systematic screening process and risk-of-bias assessment were not performed. However, efforts were made to include representative and methodologically sound studies to provide a balanced overview of the current evidence.

## 4 Tart cherries in overweight and obesity

### 4.1 Tart cherries and adipocyte inflammation–*in vitro* evidence

The potential of TC to modulate inflammatory processes associated with obesity has been preliminarily explored in several *in vitro* studies on adipocytes.

Zhou et al. (51) investigated the anti-inflammatory properties of TC extract (50 μL/mL; ~150 μg/mL anthocyanins) derived from frozen fruit in mouse adipose stem cells. Cells pre-treated with TC for 4 h and subsequently co-incubated with lipopolysaccharides (LPS; 200 ng/mL) and TC for 18 h exhibited a marked reduction in LPS-induced interleukin-6 (IL-6) level. This effect was even more prominent when TC extract and atorvastatin calcium were used together, and the reduction in IL-6 secretion was found to be greater than when the drug was applied alone.

Similarly, Jayarathne et al. (52) demonstrated that TC extract (12 μL/mL; 36 μg/mL total anthocyanins) decreased IL-6 secretion and mRNA expression of IL-6 and fatty acid synthase (FAS) in the presence of LPS (200 ng/mL) in 3 T3-L1 mouse embryo fibroblasts differentiated into adipocytes. It also increased peroxisome proliferator-activated receptor alpha (PPAR*α*) and nuclear factor erythroid-derived 2-related factor (NRF) 1–3 mRNA expression, indicated the potential impact of TC on lipid metabolism and antioxidant response in adipocytes. Nonetheless, no significant differences were observed for mRNA level of nuclear factor kappa-light-chain-enhancer of activated B cells (NFκB), tumor necrosis factor alpha (TNF-α), monocyte chemoattractant protein 1 (MCP-1) and toll-like receptors (TLR’s). Western blot analysis revealed a reduction in phosphorylated p-65 (NFκB subunit), but no significant change in phosphorylated 5′ adenosine monophosphate-activated protein kinase (p-AMPK) and phosphorylated mammalian target of rapamycin (p-mTOR) protein levels in adipocytes.

Consistent results were obtained by Harlan et al. (53), where TC extract (18–36 μg/mL) reduced IL-6 expression and secretion in 3 T3-L1 adipocytes in the presence of LPS (200 ng/mL). Moreover, TC treatment resulted in downregulation of pro-inflammatory genes such as macrophage inflammatory protein-2 (MIP2) and cyclooxygenase-2 (COX2), without affecting p-65, TLR2, TLR4, nuclear factor of activated T-cells 2 (NFAT2), or ETS transcription factor ELK1 (ELK1). TC also lowered p-65 but not c-Jun N-terminal kinase (JNK) protein expression.

Collectively, these findings suggested that TC extracts may attenuate LPS-induced inflammatory signaling in adipocytes, primarily through partial inhibition of NFκB activation and modulation of related inflammatory mediators. While these results are limited to *in vitro* models, they provided preliminary mechanistic evidence supporting the potential anti-inflammatory role of TC in obesity-related conditions.

### 4.2 Animal studies

#### 4.2.1 Rat models

Several studies have investigated the effects of TC supplementation on metabolic and inflammatory parameters in obese or high-fat diet (HFD) fed rats. One of the earliest works by Seymour et al. (54) showed that 1% (w/w) freeze-dried TC powder administered for 13 weeks to obesity-prone Zucker fatty rats fed a HFD significantly lowered serum total cholesterol, triglycerides, glucose, insulin, and pro-inflammatory cytokines (IL-6, TNF-*α*) compared with controls. TC also increased lean body mass, liver weight and decreased fat mass, retroperitoneal fat pad weight and total BW. No effect was observed on heart, kidney, and epididymal plus perirenal fat pad weight. Both the levels of TNF-α and IL-6 inflammatory cytokines as well as the mRNA expression of NFκB inhibitor alpha (IKB*α*), TNF-α and IL-6 were reduced in retroperitoneal fat, whereas an increase in the PPARα mRNA was observed in this tissue in the TC treated group. Therefore, TC supplementation was associated with reduced systemic and adipose tissue inflammation.

Consistent with these findings, Jayarathne et al. (52) reported that dietary supplementation with 4% TC powder for 8 weeks decreased IL-6, TNF-α, MCP-1, interleukin-1 *β* (IL-1β), integrin αM (CD11-b), inducible nitric oxide synthase (iNOS) and FAS mRNA levels, reduced phosphorylation of the NFκB subunit p-65, and increased anti-inflammatory marker arginase 1 (Arg-1) and antioxidant responses regulator NRF2 mRNA in epididymal adipose tissue, indicating suppression of inflammatory signaling. These effects occurred without significant changes in body weight, serum lipid profile, or glucose metabolism, suggesting that TC primarily modulates inflammatory rather than metabolic pathways in this model.

In contrast, Papp et al. (55) demonstrated that even short-term (10 days) dietary intervention with TC (0.75 g TC powder/day/rat) in male Wistar rats fed a fat-rich diet lowered total cholesterol, low-density lipoprotein (LDL) cholesterol levels, and alkaline phosphatase activity. Interestingly, this effect was significant only for two of the three cherry varieties, which were characterized by a higher total antioxidant capacity. In addition, the administration of cherries resulted in a better histological picture of the liver, including fewer lipid droplets and reduced tissue necrosis.

A comprehensive series of studies by Micioni Di Bonaventura et al. (56) examined the effects of TC seeds powder (DS; 0.1 mg/g per day) or combined TC seeds + juice (DJS; 0.1 mg/g per day + TC juice standardized to 1 mg of anthocyanins/rat per day) supplementation in HFD-induced obesity male Wistar rats over 17 weeks. TC intake reduced systolic blood pressure (SBP), glycemia, and triglyceride levels, but not BW, food intake, insulin and cholesterol levels, both in the case of rats treated with TC seeds powder as well as in combination with TC juice (DS and DJS group compared to HFD group). Researchers found no changes in the rats’ behavior as a result of the dietary intervention. However, TC intake led to increased expression of neurofilament (NF) 200 kDa in the frontal cortex and hippocampus, decreased immunoreactive microglial cells, aquaporin 4 (AQP4) and reduced area of the soma in both DS and DJS groups, while decreased astrocytes size and expression of glial fibrillary acidic protein (GFAP) was observed in DJS group. In addition, the expression of endothelial inflammatory markers, vascular cell adhesion molecule 1 (VCAM-1) and intercellular adhesion molecule 1 (ICAM-1), decreased as a result of TC intervention. Thus, although TC supplementation did not improve behavioral impairments typical of obesity, it demonstrated the ability to reduce obesity-induced neuroinflammation. In the same animals, TC supplementation attenuated hepatic steatosis, as well as increased glucose-regulated protein 94 (GRP94) level and the ratio of microtubule-associated protein light chain 3 (LC3-II/LC3-I), which may indicate a reduction in endoplasmic reticulum (ER) stress and an improvement in autophagy. TC treatment also resulted in oxidative stress attenuation, as evidenced by reduced levels of malondialdehyde (MDA) and oxidized proteins levels in the liver, as well as a decreased serum MDA levels (57).

Further analyses revealed another tissue-specific benefits. In visceral adipose tissue (VAT), TC treatment resulted in higher expression of preadipocyte factor 1 (PREF-1) mRNA, and decreased expression of cannabinoid receptor 1 (CB1), sterol regulatory element-binding protein 1c (SREBP-1c), PPARγ, uncoupling protein 2 (UCP-2), leptin, transient receptor potential vanilloid subtype 1 (TRPV1) and 2 (TRPV2) mRNA, as well as decreased expression of CB1, TRPV1 and TRPV2 proteins. Thus, it has been shown that TC may act on the endocannabinoid system by influencing the CB1, the levels of which are higher in obese individuals, and may also affect the expression of transcription factors related to lipo- and adipogenesis (SREBP-1c, PPARγ) as well as an inhibitor of adipocyte differentiation (PREF-1), and therefore improve adipose tissue functioning (58). Moruzzi et al. (59) found that TC supplementation did not affect the weight of perigonadal (PGW) or retroperitoneal (RPW) adipose tissue, but reduced the expression of inflammatory and macrophage infiltration markers. TC intake lowered C-reactive protein (CRP), cluster of differentiation 36 (CD36), TNF-*α*, IL-1β and chemokine (C-C motif) ligand 2 (CCL2) expression at the mRNA and protein levels. These findings indicate that TC supplementation attenuates inflammation and macrophage infiltration in adipose tissue. In further analyses Martinelli et al. (60) reported that TC supplementation did not affect heart weight, but reduced cardiomyocyte hypertrophy, protein and lipid oxidation. TC also downregulated key inflammatory mediators, including NFκB, platelet endothelial cell adhesion molecule-1 (PECAM-1), TNF-*α*, IL-6, ICAM-1 and IL-1β, confirming its anti-inflammatory effects in cardiac tissue. In another study by Martinelli et al. (61), the authors showed no difference in the weight of rat kidneys between TC supplemented and control groups. However, improved renal morphology, i.e., reduction of interstitial fibrosis, tubular atrophy and glomerular injury score, was observed. TC treatment significantly decreased the level of oxidized proteins and IL-6 expression in obese rats kidneys and reduced IL-1β levels. Furthermore, TC supplementation led to downregulation of transient receptor potential canonical 1 (TRPC1) and transient receptor potential melastatin 2 (TRPM2) channels, associated with renal dysfunction.

TC supplementation also appeared to beneficially affect interscapular brown adipose tissue (iBAT) in obese rats. Although iBAT weight remained unchanged, TC reduced its morphological alterations compared to control animals. Protein analyses showed increased levels of proteins involved in energy homeostasis, i.e., AMPK-*α* and p-AMPK-α, and decreased level of mitochondrial protein involved in thermogenesis, i.e., uncoupling protein-1 (UCP-1), while gene expression of PGC-1*α*, UCP-1, leptin, and adiponectin was upregulated. Despite the discrepancy between UCP-1 protein and mRNA levels, likely due to regulatory and diet-related factors, TC improved oxidative status by lowering protein oxidation and elevating GRP94, an ER stress marker. No apoptotic changes or alterations in TNF-α and IL-1β proteins were observed, although IL-6 cytokine level decreased (62). In summary, supplementation with TC seeds and juice slightly ameliorated lipid metabolism disturbances, reduced systemic and tissue-specific inflammation, lowered oxidative stress markers, and mitigated morphological alterations in the brain, liver, heart, kidneys, and adipose tissue induced by a HFD.

#### 4.2.2 Mouse models

There are only a few studies examining the effect of TC on obesity in mouse models. Nemes et al. (63) used male C57BL/6 J mice fed a HFD with 5% sucrose in drinking water and supplemented with anthocyanin-rich TC extract (60 mg/kg) for 6 weeks. Dietary intervention did not alter BW or glucose metabolism, but induced a decrease in postprandial leptin and pro-inflammatory IL-6 levels, with no effect on plasma MCP-1. It also increased water-soluble antioxidant capacity in the blood, plasma SOD and anti-inflammatory adiponectin, and reduced the level of peptide hormone resistin, which is able to promote expression of pro-inflammatory molecules as TNF-*α* and IL-6. Thus, the use of TC extract had a beneficial effect on the inflammation and oxidative status in the HFD-induced mouse model of obesity.

The same animal strain was used by Ahn et al. (64), who found that dietary TC powder supplementation (1% or 5%, 12 weeks) did not change food intake and kidney and liver weight, but decreased serum LDL, hepatic triglycerides and total cholesterol, and an increased serum high-density lipoprotein (HDL) cholesterol levels, hepatic adipose triglyceride lipase (ATGL) and hormone-sensitive lipase (HSL) activity. Treatment with a higher concentration of TC resulted in a decrease in BW, visceral fat weight, lipid droplet size in visceral fat, and lower serum triglycerides and total cholesterol levels compared to the HFD group. What’s more, TC supplementation led to modulation of hepatic protein expression, i.e., downregulation of SREBP-1c and FAS, which are proteins associated with lipid accumulation, and upregulation of p-AMPK/AMPK, phosphorylated acetyl-CoA carboxylase/acetyl-CoA carboxylase (p-ACC/ACC) and carnitine palmitoyltransferase-1 (CPT-1) proteins. Based on these results, authors suggested that the beneficial effect of regular TC consumption is related to the reduction of BW, improvement of the lipid profile and influence on hepatic lipolysis and fatty acid *β*-oxidation via the AMPK pathway.

Furthermore, in a study by Kaur et al. (65) it was shown that enriching the Western diet with TC powder (5% or 10%) for 12 weeks may affect the gut microbiota composition and increase the cecal content weight of male C57BL/6 mice. TC supplementation had a particular effect on increasing the relative abundance of phyla Firmicutes and Actinobacteria and reducing Bacteroidetes and Deferribacteres. Within the genus, dietary intervention with TC caused a decrease in *Bacteroides*, *Intestinimonas* and *Mucispirillum* and an increase in *Ruminococcaceae UCG-014*, but had no significant effect on *Lactobacillus*. These findings highlight the potential of TC to modulate the composition of the gastrointestinal microbiota.

Most studies conducted in animals used only males, limiting the ability to assess sex-specific effects. Parkman et al. (66) addressed this by using both male and female TALLYHO/Jng (TH) and C57BL/6 J (B6) mice fed a HFD supplemented with 5% TC powder for 10–14 weeks. Dietary intervention in most cases did not affect body composition, food intake, blood parameters and gonadal adipose tissue expression of adiponectin, leptin, estrogen receptor 1 and 2 genes, compared to HFD mice. However, TC increased BW and fat mass in TH females and reduced plasma IL-6 in B6 females. A more detailed gene expression analysis conducted by Seifishahpar et al. (67) revealed the effect of TC supplementation on single genes in the gonadal fat pads of the studied mice. The analysis showed TC-dependent downregulation of MCP-1 in male B6 mice, decrease in C/EBP Homologous Protein (CHOP) gene expression in male TH mice, and downregulation of Beclin1 in males of both strains, suggesting potential modulation of inflammation, ER stress, and autophagy. No changes were observed in genes related to fatty acid metabolism. The presented research indicated that not only the dose or duration of treatment, but also the strain and the sex of animals used in dietary intervention are crucial for the interpretation of the obtained outcomes. The overall results of animal studies are presented in Table 3.

**Table 3:** Main tart cherry-induced effects reported in 15 animal studies.

Reference	Animal model	Intervention	Observed outcomes
(54)	Male Zucker fatty rats fed a HFD	1% freeze-dried TC powder for 13 weeks	↓ Serum total cholesterol, triglycerides, glucose, insulin, plasma IL-6, TNF-α, % fat mass, BW, retroperitoneal fat pad weight ↓ TNF-α, IL-6, mRNA expression of IKBα, TNF-α, IL-6 in retroperitoneal fat ↓ NFkB activity in nuclear extracts of retroperitoneal fat ↑ % lean body mass, liver weight ↑ mRNA expression of PPARα in retroperitoneal fat ↔ Heart, kidney, epididymal plus perirenal fat pad weight, mRNA expression of NFκB and PPARγ in retroperitoneal fat, NFkB activity in cytosolic extracts of retroperitoneal fat
(52)	Male Zucker fatty rats fed a standard diet	4% freeze-dried Montmorency TC powder for 8 weeks	↓ mRNA expression of as IL-6, TNF-α, MCP-1, IL-1β, CD11-b, iNOS, FAS, and p-65 protein phosphorylation in epididymal fat ↑ mRNA expression of Arg-1, NRF2 in epididymal fat ↔ BW, epididymal fat pad weight, serum cholesterol, triglycerides, glucose, insulin, adiponectin, MCP-1, IL-10 ↔ mRNA expression of Egr-2, TLRs, PPARα in epididymal fat ↔ IKBα proteins and those involved in the mTOR and AMPK pathways in epididymal fat
(55)	Male Wistar rats fed a HFD	0.75 g freeze-dried TC powder from ‘Fanal’,‘Pipacs 1’ or ‘Újfehértói fürtös’ Hungarian TC varieties/day/animal for 10 days	↓ Serum total cholesterol, LDL cholesterol, alkaline phosphatase activity ↓ Lipid droplets, balloon cell degradation, vacuolization, tissue necrosis in the liver ↔ Serum HDL cholesterol, triglycerides, aspartate aminotransferase and alanine aminotransferase activity
(56)	Male Wistar rats fed a HFD	TC seeds powder at 0.1 mg/g per day or TC seeds powder (0.1 mg/g per day) + TC juice standardized to 1 mg of anthocyanins/rat per day for 17 weeks	↓ SBP, glycemia, triglycerides ↓ GFAP, immunoreactive microglial cells, AQP4, VCAM-1, ICAM-1, astrocytes size, area of soma in the frontal cortex and hippocampus ↑ NF 200 kDa in the frontal cortex and hippocampus ↔ BW, food intake, insulin, cholesterol, rats’ behavior ↔ Number of immunoreactive pyramidal neurons in the frontal cortex and hippocampus
(57)	Male Wistar rats fed a HFD	TC seeds powder at 0.1 mg/g per day or TC seeds powder (0.1 mg/g per day) + TC juice standardized to 1 mg of anthocyanins/rat per day for 17 weeks	↓ MDA, oxidized proteins in the liver, serum MDA ↓ Spleen-to-liver attenuation ratio, structural abnormalities associated with hepatic steatosis ↑ Hepatic density, GRP94, LC3-II/LC3-I ↔ SOD activity
(58)	Male Wistar rats fed a HFD	TC seeds powder at 0.1 mg/g per day or TC seeds powder (0.1 mg/g per day) + TC juice standardized to 1 mg of anthocyanins/rat per day for 17 weeks	↓ CB1, TRPV1 and TRPV2, mRNA expression of CB1, SREBP-1c, PPARγ, UCP-2, leptin, TRPV1, TRPV2 in visceral adipose tissue ↑ mRNA expression of PREF-1 in visceral adipose tissue ↔ visceral adipose tissue accumulation, HFD-induced adipocyte hypertrophy ↔ CB2, mRNA expression of adiponectin, PGC-1α, FAAH in visceral adipose tissue
(59)	Male Wistar rats fed a HFD	TC seeds powder at 0.1 mg/g per day or TC seeds powder (0.1 mg/g per day) + TC juice standardized to 1 mg of anthocyanins/rat per day for 17 weeks	↓ CRP, TNF-α, IL-1β, mRNA expression of CRP, CD36, TNF-α, IL-1β in perigonadal adipose tissue ↓ CD36, CCL2, TNF-α, mRNA expression of CD36, CCL2 in retroperitoneal adipose tissue ↔ Perigonadal and retroperitoneal adipose tissue weight
(60)	Male Wistar rats fed a HFD	TC seeds powder at 0.1 mg/g per day or TC seeds powder (0.1 mg/g per day) + TC juice standardized to 1 mg of anthocyanins/rat per day for 17 weeks	↓ Cardiomyocyte hypertrophy, protein oxidation, lipid peroxidation, NFκB, PECAM-1, TNF-α, IL-6, ICAM-1, IL-1β in the heart ↔ Heart weight, cardiac fibrosis, modulation of apoptosis, VCAM-1, E-selectin
(61)	Male Wistar rats fed a HFD	TC seeds powder at 0.1 mg/g per day or TC seeds powder (0.1 mg/g per day) + TC juice standardized to 1 mg of anthocyanins/rat per day for 17 weeks	↓ Interstitial fibrosis, tubular atrophy, glomerular injury score, protein oxidation, TRPC1, TRPM2, IL-6, IL-1β in the kidneys ↔ Kidneys weight
(62)	Male Wistar rats fed a HFD	TC seeds powder at 0.1 mg/g per day or TC seeds powder (0.1 mg/g per day) + TC juice standardized to 1 mg of anthocyanins/rat per day for 17 weeks	↓ UCP-1, IL-6, protein oxidation in interscapular brown adipose tissue ↑ p-AMPK-α, AMPK-α, GRP94, mRNA expression of PGC-1α, UCP-1, leptin, adiponectin in interscapular brown adipose tissue ↔ interscapular brown adipose tissue weight, LepR isoforms, TNF-α, IL-1β, mRNA expression of PRDM16 in interscapular brown adipose tissue
(63)	Male C57BL/6 J mice fed a HFD with 5% sucrose in drinking water	Hungarian “VN1” (selected from “Cseng˝odi csokros”) TC variety extract at 60 mg/kg for 6 weeks	↓ Postprandial leptin, IL-6, resistin ↑ Water-soluble antioxidant capacity in the blood, plasma SOD, adiponectin ↔ BW, fasting plasma glucose, glucose tolerance, postprandial plasma insulin and C-peptide, MCP-1
(64)	Male C57BL/6 J mice fed a HFD	1% or 5% TC powder for 12 weeks	↓ BW, visceral fat weight, lipid droplet size in visceral fat, serum triglycerides, total cholesterol, LDL cholesterol, hepatic triglycerides and total cholesterol, SREBP-1c, FAS in the liver ↑ Serum HDL cholesterol, hepatic adipose triglyceride lipase and hormone-sensitive lipase activity, p-AMPK/AMPK, p-ACC/ACC, CPT-1 in the liver ↔ Food intake, kidney and liver weight
(65)	Male C57BL/6 mice fed a Western diet	5% or 10% freeze-dried Montmorency TC powder for 12 weeks	↓ HOMA-IR ↑ Cecal content weight, fecal microbiota modulation ↔ BW, serum triglycerides, cholesterol, ghrelin, gastric inhibitory peptide, glucagon, PAI-1, leptin, resistin, mRNA expression of occluding, claudin, mucin 2 in the colon, zonulin-1, GPR43 in the ileum, TNF-α in the ileum lamina propria
(66)	Male and female TALLYHO/Jng (TH) and C57BL/6 J (B6), mice fed a HFD	5% freeze-dried Montmorency TC powder for 10–14 weeks	↓ IL-6 in B6 female mice ↑ BW, fat mass in TH female mice ↔ Food intake, energy expenditure, respiratory exchange ratio, locomotor activity, fasting blood glucose, glucose tolerance, mRNA expression of leptin, adiponectin, estrogen receptors 1 and 2 in gonadal adipose tissue in TH and B6 male and female mice
(67)	Male and female TALLYHO/Jng (TH) and C57BL/6 J (B6), mice fed a HFD	5% freeze-dried Montmorency TC powder for 10–14 weeks	↓ mRNA expression of MCP-1 in gonadal fat pads in B6 male mice ↓ mRNA expression of CHOP in gonadal fat pads in TH male mice ↓ mRNA expression of Beclin1 in gonadal fat pads in TH and B6 male mice ↔ mRNA expression of another inflammatory, endoplasmic reticulum stress, autophagy and fatty acid metabolism markers in gonadal adipose tissue in TH and B6 male and female mice

Arrows: ↑ increase; ↓ decrease; ↔ no effect. ACC, acetyl-CoA carboxylase; AMPK, 5′ adenosine monophosphate-activated protein kinase; Arg-1, arginase 1; AQP4, aquaporin 4; BW, body weight; CB1, cannabinoid receptor 1; CB2, cannabinoid receptor 2; CCL2, chemokine (C-C motif) ligand 2; CD11-b, integrin αM; CD36, cluster of differentiation 36; CHOP, C/EBP Homologous Protein; CPT-1, carnitine palmitoyltransferase-1; CRP, C-reactive protein; Egr-2, early growth response protein 2; FAAH, fatty acid amide hydrolase; FAS, fatty acid synthase; GFAP, glial fibrillary acidic protein; GPR43, G-protein coupled receptor 43; GRP94, glucose-regulated protein 94; HFD, high fat diet; HDL, high-density lipoprotein; HOMA-IR, homeostatic model assessment of insulin resistance; ICAM-1, intercellular adhesion molecule 1; IKBα, LepR, leptin receptor; NFκB inhibitor alpha; iNOS, inducible nitric oxide synthase; IL-10, interleukine-10; IL-1β, interleukin-1 beta; IL-6, interleukine-6; LC3, microtubule-associated protein light chain 3; LDL, low-density lipoprotein; MCP-1, monocyte chemoattractant protein 1; MDA, malondialdehyde; mTOR, mammalian target of rapamycin; NF, neurofilament; NFκB, nuclear factor kappa-light-chain-enhancer of activated B cells; NRF2, nuclear factor erythroid-derived 2-related factor 2; p-ACC, phosphorylated acetyl-CoA carboxylase; p-AMPK, phosphorylated 5′ adenosine monophosphate-activated protein kinase; PAI-1, plasminogen activator inhibitor-1; PECAM-1, platelet endothelial cell adhesion molecule-1; PGC-1α, PPARγ coactivator 1-α; PPARα, peroxisome proliferator-activated receptor alpha; PPARγ, peroxisome proliferator-activated receptor gamma; PRDM16, PR domain containing 16; PREF-1, preadipocyte factor 1; SBP, systolic blood pressure; SOD, superoxide dismutase; SREBP-1c, sterol regulatory element-binding protein 1c; TC, tart cherry; TLR, toll-like receptor; TNF-α, tumor necrosis factor alpha; TRPC1, transient receptor potential canonical 1; TRPM2, transient receptor potential melastatin 2; TRPV1, transient receptor potential vanilloid subtype 1; TRPV2, transient receptor potential vanilloid subtype 2; UCP-1, uncoupling protein-1; UCP-2, uncoupling protein 2; VCAM-1, vascular cell adhesion molecule 1.

### 4.3 Human studies

Several human trials have investigated the effects of TC supplementation on metabolic health in overweight (BMI in the range of 25.0–29.9 kg/m^2^) and obese (BMI ≥ 30 kg/m^2^) individuals. Ataie-Jafari et al. (68) reported that daily intake of 40 g concentrated TC juice for 6 weeks significantly reduced BW, diabetes indicator hemoglobin A1c (HbA1c), and SBP and diastolic blood pressure (DBP) in women with type 2 diabetes, with additional improvements in lipid profile among participants with elevated LDL baseline. This indicates that the beneficial effect of TC on the lipid profile is more unambiguous in individuals with elevated lipid parameters. In hypertensive men, Keane et al. (69) observed acute reductions in peripheral SBP and mean arterial pressure (MAP) following TC concentrate (60 mL) ingestion, accompanied by increased plasma protocatechuic and vanillic acids concentrations, which have a beneficial effect on vascular function.

Other trials in overweight and obese adults yielded mixed outcomes. In a study by Martin et al. (70) daily consumption of 240 mL of 100% TC juice for 4 weeks did not affect anthropometric indices and IL-6, IL-10, TNF-*α* and high-sensitivity CRP (hsCRP) levels, but significantly decreased MCP-1 and erythrocyte sedimentation rate (ESR), indicating a reduction in chronic inflammation. Chai et al. (71) reported that 12 weeks of TC (68 mL of concentrate in 480 mL of juice) intake lowered SBP and LDL levels, although no significant changes were observed in total cholesterol, triglycerides, HDL, insulin, or homeostatic model assessment of insulin resistance (HOMA-IR), DBP, and BW. In a complementary study (72) the authors found decreased CRP and increased 8-oxoguanine glycosylase (OGG1) levels after TC juice consumption, suggesting an improvement in oxidative stress response.

TC is considered a dietary component with a beneficial effect on uric acid levels. Martin and Coles (73) showed that TC juice (240 mL; diluted 1:6 (v/v) from concentrate) significantly reduced plasma urate in overweight and obese adults after 4-week intervention, without affecting anthropometric indices and most blood parameters. On the contrary, Stamp et al. (74) found no effect of TC concentrate on serum urate in gout patients, although, in the case of participants receiving allopurinol, urinary anthocyanin excretion increased.

In individuals with metabolic syndrome, Desai et al. (75) observed a reduction in insulin levels 1–3 h post-intake (30 mL TC concentrate in 130 mL of juice or 10 TC capsules) and a transient decrease in SBP, without affecting other cardiac haemodynamics and blood parameters. On the other hand, Johnson et al. (76) reported reductions in oxidized LDL (oxLDL) and VCAM-1, and increase in homeostatic model assessment of *β*-cell function (HOMA-%B) after 12 weeks of supplementation (240 mL of TC juice twice a day), while no changes in other blood parameters, anthropometric measurements, and hemodynamics and arterial stiffness were observed. Also, in the 1-week intervention study by Desai et al. (77) TC juice consumption (30 mL TC concentrate in 130 mL of juice) led to decreases in fasting glucose, total and LDL cholesterol, and improved resting respiratory exchange ratio (RER), 24-h DBP, 24-h SBP, 24-h MAP.

However, several studies found no significant effects of TC supplementation. No changes in anthropometric measurements, blood parameters, renal and hepatic functions were observed after a 4-week daily intake of 240 mL TC juice (78), and the dietary intervention only reduced plasma triglycerides (79). Also, in the study by Kimble et al. (80) TC juice supplementation (30 mL TC concentrate in 270 mL of juice, twice daily) for 3 months had no effects on body composition, metabolic health indices and vascular functions. Furthermore, Tucker et al. (81) did not observed any improvement in BW, inflammatory biomarkers, or objective sleep parameters following 2 weeks of 500 mg TC powder intake. The overall results of human studies are presented in Table 4.

**Table 4:** Main tart cherry-induced effects reported in 14 human studies.

Reference	Study design	Subjects	Intervention	Observed outcomes
(68)	Quasi-experimental study	19 diabetic (type 2) women (BMI 29.6 ± 4.3 kg/m^2^, age 53.6 ± 8.8 years)	40 g concentrated TC juice daily for 6 weeks	↓ BW, HbA1c, SBP, DBP ↓ Total cholesterol, LDL cholesterol, in the subgroup of 12 participants with a baseline of LDL ≥ 100 mg/dL ↔ Fasting blood sugar, HDL cholesterol, triglycerides
(69)	Placebo-controlled, blinded, crossover, randomized Latin square design study	15 nonsmoking men with early hypertension (BMI 27.0 ± 3.8 kg/m^2^, age 31 ± 9 years)	60 mL Montmorency TC concentrate acutely	↓ SBP, MAP ↑ Plasma concentrations of protocatechuic and vanillic acids ↔ DBP, microvascular vasodilation and other vascular variables, plasma nitrate and nitrite
(70)	Randomized, placebo-controlled crossover study	10 participants (80% females; BMI 32.2 ± 4.6 kg/m^2^, age 38.1 ± 12.5 years)	240 mL 100% TC juice daily for 4 weeks	↓ MCP-1, ESR ↔ BW, IL-6, IL-10, TNF-α, hsCRP
(71)	Parallel, randomized controlled study	TC group: characteristic of 20 initial participants, 17 of whom completed: 60% females, BMI 28.5 ± 3.7 kg/m^2^, age 70.0 ± 3.7 years; placebo group: 17 participants, 47% females, BMI 27.3 ± 4.2 kg/m^2^, age 69,5 ± 3.9 years	240 mL Montmorency TC juice (prepared from 34 mL of concentrate) twice a day for 12 weeks	↓ SBP, LDL cholesterol ↑ Glucose ↔ BW, DBP, total cholesterol, triglycerides, HDL cholesterol, insulin, HOMA-IR
(72)	Parallel, randomized controlled study	TC group: characteristic of 20 initial participants, 17 of whom completed: 60% females, BMI 28.5 ± 3.7 kg/m^2^, age 70.0 ± 3.7 years; placebo group: 17 participants, 47% females, BMI 27.3 ± 4.2 kg/m^2^, age 69,5 ± 3.9 years	240 mL Montmorency TC juice (prepared from 34 mL of concentrate) twice a day for 12 weeks	↓ CRP ↑ OGG1 ↔ TNF-α and other biomarkers of inflammation and oxidative stress
(73)	Randomized, placebo-controlled crossover study	26 overweight and obese participants (69% females, BMI 31.3 ± 6.0 kg/m^2^, age 41 ± 11 years; 12 of them displayed hyperuricemia)	240 mL TC juice (diluted 1:6 (v/v) from concentrate) daily for 4 weeks	↓ Plasma urate ↔ BMI, SBP, DBP, ESR, total cholesterol, LDL cholesterol, HDL cholesterol, triglycerides, glucose, insulin, hsCRP, MCP-1, HOMA
(74)	Short term, dose ranging, time course trial	Participants with gout (characteristics of 50 initial participants, 46 of whom completed: 10% females, average BMI 28.8–31.2 kg/m^2^, average age 56.2–63.3 years; 25 participants on allopurinol and 25 on no urate-lowering therapy)	5 different Montmorency TC concentrations groups: i.e. placebo 2 drops, 7.5 mL twice daily, 15 mL twice daily, 22.5 mL twice daily, 30 mL twice daily in 250 mL water for 28 days	↑ Urinary anthocyanins in participants receiving allopurinol ↔ BW, serum urate, SBP, DBP, HbA1c
(75)	Single-blind, placebo-controlled, randomised, crossover study	11 participants (45% females, BMI ~ 33 kg/m^2^, age 49 ± 12 years) with metabolic syndrome	130 mL Montmorency TC juice (prepared from 30 mL of concentrate) or 10 Montmorency TC capsules, acutely	↓ Insulin, SBP ↔ Glucose, triglycerides, total cholesterol, HDL cholesterol, LDL cholesterol, other cardiac haemodynamics and arterial stiffness parameters
(76)	Randomized, single-blind, placebo-controlled, parallel-arm pilot clinical trial	10 participants in placebo group: 50% females, BMI 33.5 ± 1.8 kg/m^2^, age 44.2 ± 4.1 years; 9 participants in TC group: (44% females, BMI 34.3 ± 1.9 kg/m^2^, age 29.3 ± 1.1 years); participants with metabolic syndrome	240 mL of Montmorency TC juice twice a day for 12 weeks	↓ Oxidized LDL cholesterol, VCAM-1 ↑ HOMA-%B ↔ BW, haemodynamics and arterial stiffness parameters, ICAM-1, glucose, insulin, total cholesterol, triglycerides, HDL cholesterol, LDL cholesterol, leptin, adiponectin, HOMA-IR
(77)	Single-blind, placebo-controlled, randomized, crossover study	6 males and 6 postmenopausal females with metabolic syndrome (BMI 31 ± 7 kg/m^2^, age 50 ± 10 years)	130 mL Montmorency TC juice (prepared from 30 mL of concentrate) daily for 1 week	↓ Fasting glucose, total cholesterol, LDL cholesterol, RER, 24-h DBP, 24-h SBP, 24-h MAP ↔ Insulin, triglycerides, HDL cholesterol, other cardiac hemodynamics and arterial stiffness parameters
(78)	Randomized, crossover, placebo-controlled study	10 participants (80% females, BMI 32.2 ± 4.6 kg/m^2^, age 38.1 ± 12.5 years)	240 mL 100% TC juice daily for 4 weeks	↔ BW, glucose, hepatic enzymes in plasma, renal functions biomarkers in plasma
(79)	Randomized, crossover, placebo-controlled study	10 participants (80% females, BMI 32.2 ± 4.6 kg/m^2^, age 38.1 ± 12.5 years)	240 mL 100% TC juice daily for 4 weeks	↓ Triglycerides ↔ Insulin, total cholesterol, HDL cholesterol, LDL cholesterol
(80)	Randomized, placebo-controlled, double-blind parallel study	50 participants (32% females, BMI 27.6 ± 3.7 kg/m^2^, age 48 ± 6 years)	270 mL Montmorency TC juice (prepared from 30 mL of concentrate) twice daily for 3 months	↔ BW, vascular function, glucose, insulin, HOMA-IR, hsCRP, triglycerides, total cholesterol, HDL cholesterol, LDL cholesterol
(81)	Randomized, controlled, crossover study	34 participants with sleep issues (75% females, BMI 32.1 ± 7.0 kg/m^2^, age 32.6 ± 10.7)	500 mg dose of 100% Montmorency TC powder in the form of two pills one hour before bedtime daily for 2 weeks	↓ Subjective sleep parameters (Insomnia Severity Index, Pittsburgh Sleep Quality Index) compared to baseline ↔ BW, inflammatory biomarkers, objective sleep outcomes

Arrows: ↑ increase; ↓ decrease; ↔ no effect. CRP, C-reactive protein; DBP, diastolic blood pressure; ESR, erythrocyte sedimentation rate; HbA1c, hemoglobin A1c; HDL, high-density lipoprotein; HOMA-IR, homeostatic model assessment of insulin resistance; HOMA-%B, homeostatic model assessment of β-cell function; hsCRP, high-sensitivity CRP; ICAM-1, intercellular adhesion molecule 1; IL-10, interleukine-10; IL-6, interleukine-6; LDL, low-density lipoprotein; MAP, mean arterial pressure; MCP-1, monocyte chemoattractant protein 1; OGG1, 8-oxoguanine glycosylase; RER, resting respiratory exchange ratio; SBP, systolic blood pressure; TC, tart cherry; TNF-α, tumor necrosis factor alpha; VCAM-1, vascular cell adhesion molecule 1.

## 5 Discussion and conclusion

The results of the presented studies provide growing, although still inconsistent, evidence regarding the potential of TC supplementation in the context of obesity and related metabolic disturbances. Most findings from animal studies indicate beneficial effects of TC, particularly in reducing oxidative stress and inflammation markers across several tissues, including adipose tissue, liver, and heart. The reported improvements in antioxidant status and modulation of cytokines such as IL-6 or TNF-*α* are in line with observations for other anthocyanin-rich fruits, including blueberries (82, 83), chokeberries (84, 85), pomegranates (86), elderberries (87), or grapes (88, 89). However, TC dietary intervention yielded inconsistent results with respect to body weight, lipid metabolism, and glucose homeostasis, suggesting that TC exerts primarily modulatory rather than corrective actions on metabolic outcomes.

Human studies, although more heterogeneous, tend to confirm the anti-inflammatory and blood pressure-lowering properties of TC supplementation in overweight and obese individuals, while showing little or no effect on lipid profile, insulin sensitivity, or body composition. This pattern of effects is comparable to that described for other polyphenol-rich foods, where moderate improvements in vascular function and oxidative status are more consistently reported than changes in lipid profile or glycemic control, in both healthy and metabolically diseased subjects (90–92). It is worth noting that broader meta-analyses including both healthy and metabolically impaired participants, despite some discrepancies in overall findings, suggest that the potential benefits of TC on glycemic and lipid parameters may be more pronounced in some specific subgroups, i.e., obese, elderly and/or metabolically compromised individuals (93, 94). Notably, the reduction in systolic blood pressure observed in several trials supports the hypothesis that polyphenols in TC may enhance endothelial function and nitric oxide bioavailability, as was already shown for different anthocyanins, their metabolites, and anthocyanins-rich fruits and extracts (95, 96).

**Figure 1:**
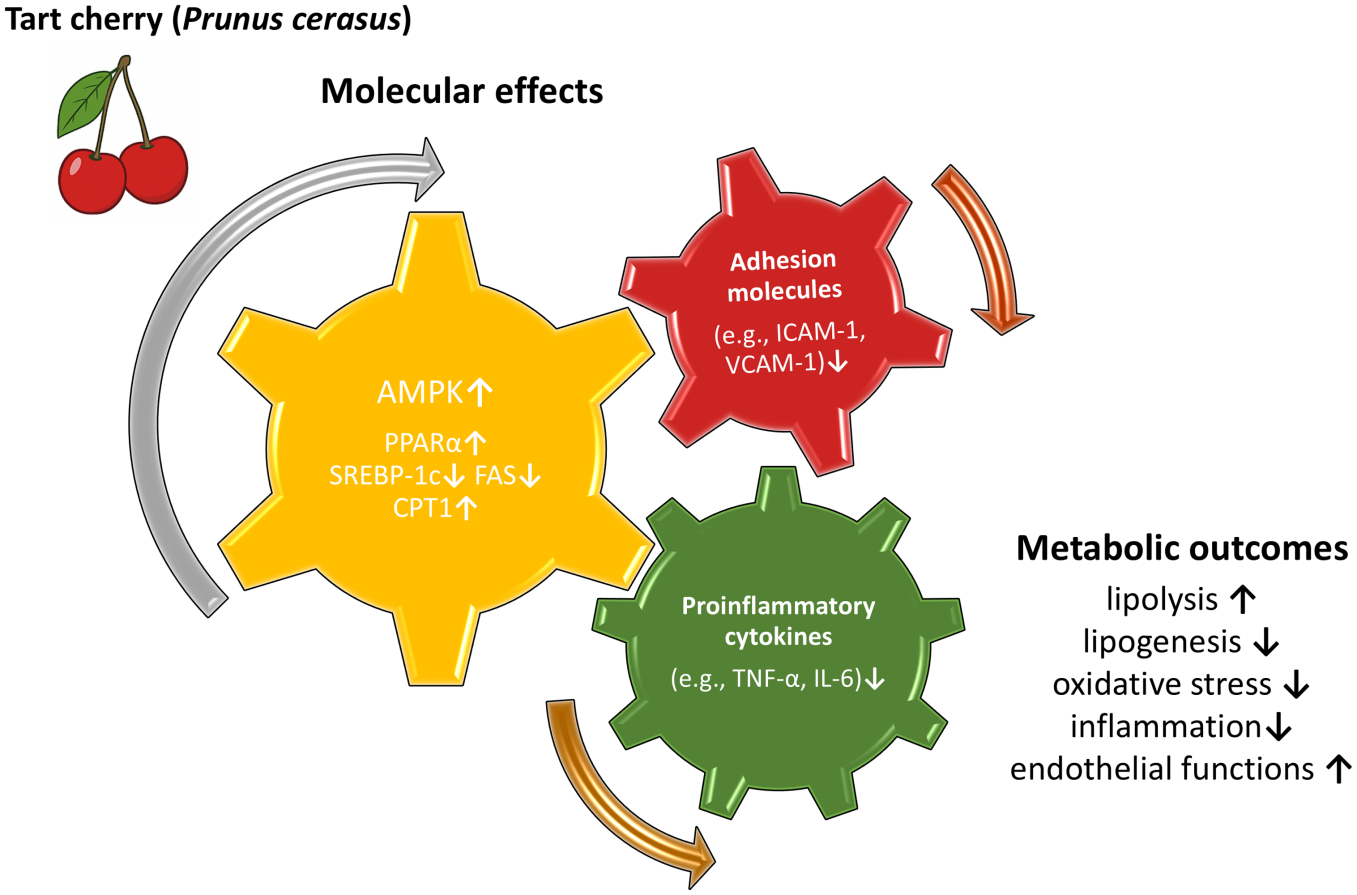
Proposed mechanisms underlying the effects of tart cherry consumption on metabolic health. Tart cherry-derived bioactive compounds may activate AMPK and modulate lipid metabolism-related pathways (e.g., PPAR*α*, SREBP-1c, FAS, CPT1), while reducing proinflammatory cytokines (e.g., TNF-α, IL-6) and adhesion molecules (e.g., ICAM-1, VCAM-1). These effects may contribute to improved metabolic outcomes, including increased lipolysis, reduced lipogenesis, attenuation of oxidative stress and inflammation, and improved endothelial functions.

From a mechanistic perspective, some evidence from *in vitro* and *in vivo* studies suggests that the AMPK pathway and genes regulating lipid metabolism, such as SREBP-1c, FAS, and CPT-1, may contribute to the observed effects of TC. These mechanisms resemble those proposed for other flavonoid-rich foods. Valenti et al. (97) suggested that anthocyanins may exert beneficial effects in nonalcoholic fatty liver disease by downregulating SREBP-1c and thereby limiting lipogenesis, enhancing PPARα-mediated lipolysis, and upregulating antioxidant enzymes, which appear to be connected with AMPK pathway activation. Similar mechanisms have also been reported in adipose tissue in obesity studies involving both anthocyanins and other flavonoids, where these compounds were shown to activate the AMPK pathway and modulate mitochondrial biogenesis, oxidative stress, and inflammation (98, 99). These findings suggest that TC may act through shared molecular targets regulating lipid metabolism, mitochondrial function, and inflammatory signaling. Proposed mechanisms underlying the effects of TC are presented in the Figure 1. Future studies integrating metabolomic and transcriptomic approaches could help clarify the molecular interactions underlying the physiological responses to TC intake.

Although the proposed mechanisms support the biological plausibility of TC’s health effects, discrepancies among studies indicate that these outcomes may be strongly influenced by methodological and biological factors. Differences in experimental design, including the duration of supplementation, the form of TC used (juice, powder, extract, capsules), dose, concentration, and stability of bioactive compounds, as well as the animal strain, likely contribute to the heterogeneity in reported results. In addition, variability in the compositional characterization of TC products represents an important but often overlooked source of inconsistency. In many studies, only total anthocyanin and total phenolic content is reported, while detailed profiling of individual compounds, extraction procedures, and analytical methodologies are frequently lacking, particularly in human interventions using commercially available products. This limits direct comparability between studies and complicates the identification of specific bioactive constituents responsible for the observed effects. The anthocyanin content of TC products varies considerably depending on cultivar, processing, and storage, which in turn affects bioavailability and physiological outcomes. Furthermore, most animal studies were conducted using male rodents, limiting the ability to identify sex-specific responses. Similarly, in human interventions, differences in baseline health status, habitual diet, and physical activity may confound the metabolic response to TC supplementation. These limitations highlight the need for standardized protocols, improved compositional characterization, and well-characterized interventions to enable meaningful cross-study comparisons.

In summary, TC represents a promising dietary component with the potential to alleviate low-grade inflammation and oxidative stress associated with obesity. Nevertheless, evidence regarding its influence on lipid metabolism, glucose regulation, and body weight remains inconsistent. While the inclusion of TC in the diet may contribute to cardiovascular and metabolic health, the magnitude of these effects seems modest and context-dependent, therefore, the outcomes of the presented studies should be interpreted with caution. Future research should aim to identify the specific bioactive compounds and metabolites responsible for the observed effects, as well as to better understand their bioavailability and interactions within the food matrix. An important area that remains to be explored is the interindividual variability in response to TC supplementation, including the role of sex, gut microbiota, and baseline metabolic status. From an application perspective, further work is needed to determine optimal forms, doses, and processing methods that preserve bioactivity and maximize efficacy. Such insights could support the development of targeted functional foods or nutraceutical formulations designed to promote metabolic health and assist in body weight management.
